# The Importance of Nasal Breathing in Early Childhood*Read at the annual meeting of the West Texas Medical Society, San Antonio, October 26, 1899.

**Published:** 1899-11

**Authors:** R. E. Moss

**Affiliations:** San Antonio, Texas


For Texas Medical Journal.

### The Importance of Nasal Breathing in Early Childhood.*

BY R. E. MOSS, M. D., SAN ANTONIO, TEXAS.

The subject of this short paper would be, seemingly, like stating a self-evident proposition, and I feel almost as if I should apologize for doing so. It would seem that every intelligent thinking man would appreciate it, but there are many children today, even in well to do families, whose parents treat the affliction as a careless habit on the part of the child or else are using a spray in an indifferent, irregular way, expecting results or that the child will "outgrow" the habit. Imperfect nasal breathing is injurious to adults as well, but I wish to limit my remarks to children, for the neglect here is far-reaching and the results brilliant when properly treated. It would be well just here to study the purpose or physiological function of the nose. As we know anatomically there are the three turbinated bodies on each side with the accessory sinuses, namely, antrum, etmoid, frontal and sphenoid. In the nasopharynx there is a lymphoid or glandular mass known as the third tonsil or adenoids, and on either side of this space the opening to each Eustachian tube. The turbinated bodies are richly supplied with nerves, blood-vessels and mucous glands, and by their peculiar scroll shape affording a large surface for the inspired air to come in contact with. The air is thus warmed and moistened and to a great extent freed from dust before entering the lungs.

The importance of this being properly done is far reaching in its benefits, mentallv and physically. It is impossible, in a paper of this kind, to do more than treat the subject in a general way; therefore, I shall not enter into many important details. General development we find, as a rule, very much below the average for children of same age not similarly afflicted. They take cold easily, have capricious appetites, frequently chronic cough, flat chest, tendency to stoop or become round shouldered. They have heavily coated tongues, foul breath, suffer from malassimilation of food, and as a rule are constipated when not suffering from diarrhœa. The result of this, is lowered vitality and a predisposition to disease. These cases, when attacked by some acute disease, oftentimes succumb or suffer from complications that another would have escaped.

The secretion of a healthy nose, according to late bacteriological

---

*Read at the annual meeting of the West Texas Medical Society, San Antonio, October 26, 1899.

investigations, possesses germicidal properties to a certain extent, and it acts more or less as a sieve in ridding the air of dust, and necessarily of the germs clinging to same. It is not fanciful or theoretical to say that this is of the greatest possible value to each individual possessing a healthy nose. These children not only suffer from lack of bodily development, but, as can be easily proven, the mental condition is in extreme cases pitiable indeed, even bordering upon idiocy. The dull, expressionless face of a mouth-breathing child is familiar to everyone, but there are numbers who are occassional mouth breathers or only at night when asleep, and, of course, we find less mental dullness. Who can estimate the effect upon the general welfare of the child, even granting he escapes the numerous complications that such a condition predisposes him to? Just how the mind is affected by partial or complete nasal occlusion is not definitely settled, nor does it matter to us clinically. Experiments have shown that where one side of the nose in young animals has been sewed up the brain on same side has failed to develop as much as the opposite side. Each of us knows how dull we feel when suffering from cold which causes marked congestion of nose, particularly of superior and middle turbinated bones. If we consider the construction of the cribriform plate of ethmoid bone, with its numerous openings for blood-vessels passing directly from meninges to nose and from nose to meninges, it would seem, as a recent writer claimed, for the purpose of aerating the blood and carrying oxygen to the brain. This is like many unsolved problems, but fortunately we can remedy the evil if proper measures are adopted sufficiently early. This condition of the nose is the direct cause of the greater number of ear troubles with children, from the slight earache to suppurative otitis, both acute and chronic, including mastoid complications.

There are a host of deaf people in the world today who owe their deplorable condition to neglect in early life of this important function. I could report scores of cases where the hearing was reduced to firm pressure with a watch against the ear, and after operation, followed by judicious treatment, the watch could be heard three to four feet away. These cases do not get well without treatment nor do they "outgrow it," as so many anxious mothers have been told by the family physician. It is true that the tonsils atrophy about the age of puberty and the mouth breathing may be relieved, but not so with the defective hearing. This condition also causes disease of the accessory sinuses and affects phonation in a very marked manner. There is a lack of proper development of the bones of the nose and palate particularly, and after a few years, no matter how correctly treated, there is always that nasal intonation of voice so disagreeable to listen to. Deafmutism can correctly be charged to this condition in many instances.

Infants are frequently affected with adenoids causing direct pressure upon the Eustachian tubes, thereby rendering the child partially or completely deaf long before it begins to talk. You readily understand such a child would be a deaf mute if not treated very early. This condition of the nose is directly causative of many reflex disturbances, as well as some of the more important neuroses.

Three of the most frequent as well as most important are chronic cough, enuresis, and chorea. I have records of quite a number of interesting cases suffering from above conditions that were entirely cured by establishing free nasal breathing. These cases had been intelligently treated, from the standpoint of tonics and hygiene, from a few months to several years, without more than temporary benefit. I do not wish to create the impression that all the ills of childhood can be cured or prevented by the nose and throat specialist, but I do earnestly ask that the general practitioner will pay particular attention to this condition in the early years of childhood. There is no condition in life where the old adage, "an ounce of prevention is worth a pound of cure," is more applicable. I am in the habit of giving parents this simple rule to guide them as to when they should bring their children to me for examination or treatment: If the child is very restless at night, or breathes through the mouth when asleep, inattentive when spoken to, or suffers with recurring attacks of earache. I shall not enter upon the treatment at this time; my remarks have been principally for the purpose of directing the attention of the general practitioner to this important and oftentimes sadly neglected function of early life. If I have succeeded in this regard my efforts have been well repaid.

